# Supplementary material for: Outcomes of primary rhegmatogenous retinal detachment repair with extensive scleral-depressed vitreous removal and dynamic examination
Source: PLoS One. 2020 Sep 24;15(9):e0239138. doi: 10.1371/journal.pone.0239138 (PMC7514044; doi:10.1371/journal.pone.0239138)
Supplement: Attachment — Submitted filename: Response to Reviewers.docx [file pone.0239138.s002.docx]

Manuscript Number: PONE-D-20-19374
Title: Outcomes of Primary Rhegmatogenous Retinal Detachment Repair with Extensive Scleral-Depressed Vitreous Removal and Dynamic Examination
PLOS ONE

Response to Reviewers

August 4, 2020

Dear Dr. Kashani,

We would like to sincerely thank the reviewers for their scholarly comments regarding our manuscript entitled "Outcomes of Primary Rhegmatogenous Retinal Detachment Repair with Extensive Scleral-Depressed Vitreous Removal and Dynamic Examination". We believe that with their suggestions they have helped us improve our manuscript considerably.

With best regards,

John B Miller MD

Corresponding Author

 Response to Editorial Issues

*1. Please ensure that your manuscript meets PLOS ONE's style requirements, including those for file naming. The PLOS ONE style templates can be found at*

*https://journals.plos.org/plosone/s/file?id=wjVg/PLOSOne_formatting_sample_main_body.pdf &https://journals.plos.org/plosone/s/file?id=ba62/PLOSOne_formatting_sample_title_authors_affiliations.pdf*

 We thank the editor for pointing out the subtle changes in style requirement. Based on the 2 above PDFs, we have changed the formatting to appropriately match the formatting guidelines.

2. *We note that you have indicated that data from this study are available upon request. PLOS only allows data to be available upon request if there are legal or ethical restrictions on sharing data publicly. For information on unacceptable data access restrictions, please see http://journals.plos.org/plosone/s/data-availability#loc-unacceptable-data-access-restrictions.*

*In your revised cover letter, please address the following prompts:*

*a) If there are ethical or legal restrictions on sharing a de-identified data set, please explain them in detail (e.g., data contain potentially identifying or sensitive patient information) and who has imposed them (e.g., an ethics committee). Please also provide contact information for a data access committee, ethics committee, or other institutional body to which data requests may be sent.*

*b) If there are no restrictions, please upload the minimal anonymized data set necessary to replicate your study findings as either Supporting Information files or to a stable, public repository and provide us with the relevant URLs, DOIs, or accession numbers. Please see http://www.bmj.com/content/340/bmj.c181.long for guidelines on how to de-identify and prepare clinical data for publication. For a list of acceptable repositories, please see http://journals.plos.org/plosone/s/data-availability#loc-recommended-repositories*.

We will submit an anonymized data set as a Supporting Information file.

 Response to Reviewers

*Reviewer #1: Nice article adding to the body of literature demonstrating the high success rate with vitrectomy and emphasizing the importance extensive shaving practices in achieving high successes. The only criticism would be as the authors alluded to of the high percentage of caucasian's in the cohort.*

We thank the reviewer for this comment; and agree that our patient population may not match that of another institution or of a private practice vitreoretinal surgeon. We have added the following at line 333:

“... (patient population **[particularly age or ethnicity**]…”

*It would also be nice for the authors to emphasize that while success rate are extremely good at 3 month followup, the authors need to more explicitly state that longer term outcomes > 6 months is unclear due to 8.5% of patients lost to followup.*

We thank the reviewer for their close reading and agree that an explicit differentiation between outcomes at 3 months and 1 year should be discussed. We had added the following at line 336:

“…6 months of follow up. **Thus, while the surgical success rate was high at 3 months and remained high at 1 year, the exact success rate at 1 year may be lower since some eyes received follow up care elsewhere**.”

Reviewer #2:

*In this paper the authors present Outcomes of over 180 consecutive Primary Rhegmatogenous Retinal Detachment Repairs with Extensive Scleral-Depressed Vitreous Removal and Dynamic Examination.
The paper is well written and the content is appropriate and of interest to vitreoretinal surgeons. Their method described is of interest due to the high anatomic success rate. I have the below comments which may help clarify something of the points raised in the paper:*

*1.Just over 15% of eyes had a scleral buckle. The use of scleral buckle and case selection is of interest. Some general comments were made regarding which cases had this procedure done. More specific case selection would be of interest. For example, all patients with inferior pathology or with multiple breaks and inferior pathology or with total detachment and inferior pathology etc had a buckle. Since this is not a small percentage of patients, if the use of buckle was based on one surgeon’s preference or some combination of pathologies, that would be important and useful information to share.*

We thank the reviewer for the warm positive comments. We also appreciate diving into the finer details, such as the use of adjuvant scleral buckle (SB). All 3 surgeons had equivalent rates of SB (17.5% vs 15.5 vs 13.6 %). SB was used in 6 out of 24 (25%) eyes with PVR, 7 out of 17 eyes (41%) with inferior breaks, 17 of 68 (25%) superior & inferior breaks, and 2 of 3 (66%) eyes with no preoperative break seen. Patients that received SB were also younger; 20 of 29 eyes (69%) were less than 60 years old, and 10 eyes (34.5%) were between the ages of 30-50.

We made reference to these guidelines in line 165-166. We have added the following at line 166:

**“All 3 surgeons had equivalent rates of adjuvant SB use. SB was implemented in younger patients (20 of 29 eyes [69%] were less than 60 years old, and 10 eyes [34.5%] were between the ages of 30-50), detachments with inferior breaks (24 out of 85 [28%]), and eyes with PVR [6 out of 24 (25%)]”**

.

*Over 40% of patients had triamcinolone used in the eye to assist in vitrectomy. That is also relatively a high percentage of eyes that had this technique utilized. Was this only used for vitreous shaving based on poor view to the periphery from lens opacity or was it also utilized to elevate the hyaloid or evaluate for the presence of complete posterior vitreous detachment. The criteria of use or whether this was used mostly by one surgeon over others will be useful.*

We agree with the reviewer that our triamcinolone (TA) use may not be similar to other surgeons. As written in line 166-168, TA was used to stain remaining cortical vitreous in phakic eyes with a hazy peripheral view from different anterior segment pathology in order to shave all identifiable vitreous. TA was infrequently used to elevate the hyaloid or confirm the presence of a posterior vitreous detachment (PVD) as >90% of eyes in this cohort had a naturally occurring PVD. We have added the following at line 339:

**“In addition, triamcinolone was used in ~40% of cases, when the far peripheral view was obscured (e.g. due to cataract, capsular phimosis, or other anterior segment media opacities) in order to identify any residual vitreous for subsequent removal.”**

*There were a few eyes that were Aphakic at last follow up. Was pars plana lensectomy performed in these eyes during the primary vitrectomy? What was the criteria used to perform the lensectomy.*

We thank the reviewer for this comment. The 3 cases of aphakia were 1 patient with Marfan’s syndrome with a history of bilateral lens dislocation who was aphakic when they presented to us (line 189) and 1 patient with unilateral juvenile cataract since age of 2 that was also was left aphakic (line 189-190). To provide some clarity, we have added the following at line 213:

“…**who had a remote history of bilateral lens dislocation (left aphakic OU)**…”

*The active flow used to identify peripheral breaks has not been clearly described in the methods section. This was a technique that the authors credit in their high success rate. A
Better description of how this was used would be helpful. What flow rate or approximate percentage flow rate is used? What is the cut rate of the vitrectomy during this technique. Is that different than the vitreous shaving technique or performed while shaving the vitreous under active scleral depression? What percentage of breaks (in cases of multiple breaks) were identified with this technique (if known)?*

We thank the reviewer for their close reading. We have modified the manuscript to provide more details about the vitrectomy settings and dynamic examination technique as indicated below. We did not record the percentage of breaks identified with this technique. We have added the following at line 175:

***“*A dynamic examination of the far periphery using active flow with the cutter was used to identify additional breaks. At the time of this study, a high cut rate of 7500 cpm and 650 mmHg for vacuum was commonly used to induce elevation of small retinal tear flaps and suspicious lesions within the sphere of influence of the vitreous cutter. In our experience, this helped identify small retinal breaks not seen pre-operatively or with scleral depression alone.”**

*All eyes had focal laser performed to identified pathology, yes three eyes had no breaks identified. What was Pathology was identified and lasered in these eyes?*

We thank the reviewer for their close reading. We have added the following at line 239:

“ **In the 2 of the 3 eyes with no identifiable intra-operative retinal breaks, a scleral buckle was placed, and laser was performed around the drainage retinotomy site as well as the posterior edge of the scleral buckle. In the 3^rd^ eye, additional laser was performed around a previous break that was previously barricaded by laser retinopexy.”**
